# Supplementary material for: Idiopathic and secondary restless legs syndrome during pregnancy in Japan: Prevalence, clinical features and delivery-related outcomes
Source: PLoS One. 2021 May 11;16(5):e0251298. doi: 10.1371/journal.pone.0251298 (PMC8112660; doi:10.1371/journal.pone.0251298)
Supplement: S1 Fig — (PDF) [file pone.0251298.s001.pdf]

Supplementary Figure 1. Clinical course and use of the medications in all cases of restless legs syndrome

	Pre-Pregnancy	Second trimester	Third trimester	After delivery
Idiopathic RLS				
Case 1				
Diagnosis	○	○	Recovered	Recovered
Iron			↔	
Folate		↔		
Multivitamin	↔			
Case 2				
Diagnosis	○	○	Recovered	Recovered
Iron		↔	↔	↔
Folate		↔	↔	↔
Multivitamins		↔		↔
Case 3				
Diagnosis	○	○	Recovered	Recovered
Iron		↔	↔	
Folate		↔	↔	↔
Case 4				
Diagnosis	○	○	Recovered	Recovered
Iron		↔	↔	
Folate		↔	↔	
Multivitamins			↔	
Case 5				
Diagnosis	○	○	Recovered	Recovered
Iron		↔	↔	
Folate		↔		
Case 6				
Diagnosis	○	○	Recovered	Recovered
Folate			↔	
Case 7				
Diagnosis	○	Recovered	Recovered	Recovered
Folate		↔		

Multivitamins		↔	↔	↔
Case 8				
Diagnosis	○	Recovered	Recovered	Recovered
Folate		↔		
Case 9				
Diagnosis	○	Recovered	Recovered	Recovered
Iron			↔	
Multivitamins				↔
Secondary RLS onset at 2nd trimester				
Case 1				
Diagnosis	None	○	○	Recovered
Case 2				
Diagnosis	None	○	Recovered	Recovered
Folate		↔	↔	
Case 3				
Diagnosis	None	○	Recovered	Recovered
Iron			↔	
Folate		↔		
Secondary RLS onset at 3rd trimester				
Case 1				
Diagnosis	None	None	○	Recovered
Folate	↔	↔	↔	
Multivitamins	↔	↔	↔	
Case 2				
Diagnosis	None	None	○	Recovered
Iron		↔		
Folate	↔	↔		
Case 3				
Diagnosis	None	None	○	Recovered
Iron		↔	↔	
Case 4				
Diagnosis	None	None	○	Recovered
Iron			↔	
Folate			↔	↔

Case 5				
Diagnosis	None	None	○	Recovered
Iron			↔	
Case 6				
Diagnosis	None	None	○	Recovered
Iron				↔
Case 7				
Diagnosis	None	None	○	○
Iron			↔	↔

RLS: restless legs syndrome

○: Diagnosis of RLS.
