# Supplementary material for: Idiopathic and secondary restless legs syndrome during pregnancy in Japan: Prevalence, clinical features and delivery-related outcomes
Source: PLoS One. 2021 May 11;16(5):e0251298. doi: 10.1371/journal.pone.0251298 (PMC8112660; doi:10.1371/journal.pone.0251298)
Supplement: S1 Table — (PDF) [file pone.0251298.s002.pdf]

Supplementary Table1. Relationship of maternal age, body mass index, hypertension, gestational diabetes mellitus and endometriosis with Perinatal RLS

	Perinatal RLS n (%)	P value
Maternal Age		
<30 years (n=52)	3 (5.8%)	
≥30 years (n=130)	16 (12.3%)	0.19
Body mass index		
<20 kg/m^2 (n=23)	2 (8.7%)	
≥20 kg/m^2 (n=159)	17 (10.7%)	0.77
Hypertension		
Absent (n=181)	19 (10.5%)	
Present (n=1)	0 (0.0%)	0.73
Gestational diabetes		
Absent (n=181)	19 (10.5%)	
Present (n=1)	0 (0.0%)	0.73
Endometriosis		
Absent (n=179)	19 (10.6%)	
Present (n=3)	0 (0.0%)	0.55

P values were estimated using the chi-squared tests.

RLS: Restless legs syndrome
