# Supplementary material for: Idiopathic and secondary restless legs syndrome during pregnancy in Japan: Prevalence, clinical features and delivery-related outcomes
Source: PLoS One. 2021 May 11;16(5):e0251298. doi: 10.1371/journal.pone.0251298 (PMC8112660; doi:10.1371/journal.pone.0251298)
Supplement: Attachment — Submitted filename: Response to Reviewers revise2021.4.21.docx [file pone.0251298.s004.docx]

**Response to Reviewer #2**

Thank you for your useful suggestions.

We have attempted to address your suggestions as follows:

***Major comment 1.*** *In response to my primary question about the study, the authors state that "RLS did improve for many of the subjects in our study." However that under-emphasizes this finding and perhaps misses the point I was trying to make. While some studies have reported improvement in RLS symptoms during pregnancy for a minority of subjects with pre-existing RLS (11% on systemic review doi:10.1186/s12883-020-01709-0), to my knowledge none have reported complete resolution of RLS in such large number of cases.*

**Response:**

As you pointed out, our findings were different from those from previous publications. While, in our study, idiopathic RLS in the 2^nd^ trimester disappeared in the 3^rd^ trimester, a Chinese cross-sectional study reported higher prevalence of idiopathic RLS in the 3^rd^ trimester than in the 2^nd^ trimester. Another longitudinal study conducted in Italy reported comparable prevalence rates but 11% of pregnant women with idiopathic RLS reported improvement of symptoms. The reasons for various time courses in prevalence of idiopathic RLS in 2^nd^ and 3^rd^ trimesters are not clear but discrepancies might be attributable to differences in study design (longitudinal or cross-sectional), ethnicity, use of medications (iron, folic acid and multivitamins), susceptibility to medications etc. We have discussed this point in the 3^rd^ paragraph of the Discussion (line 3-17, page 12) as follows.

“In the present analysis, prevalence of idiopathic RLS decreased from 3.3% in the 2^nd^ trimester to 0% in the 3^rd^ trimester. There has still be limited publications which reported trend in prevalence of RLS by type (idiopathic vs secondary) from 2^nd^ to 3^rd^ trimester. An observational study of 1584 pregnant women in China reported higher prevalence of idiopathic RLS in 3^rd^ trimester (5.2%) than in 2^nd^ trimester (4.3%) while this was a cross-sectional study and did not follow participants from 2^nd^ to 3^rd^ trimester. Another longitudinal study of 642 pregnant women in Italy reported comparable prevalence rates of idiopathic RLS between 2^nd^ and 3^rd^ trimesters but approximately 11% of participants with idiopathic RLS in the 2^nd^ trimester showed improvement in RLS symptoms in the 3rd trimester. The reasons for various time courses in prevalence of idiopathic RLS in 2^nd^ and 3^rd^ trimesters are not clear but discrepancies might be attributable to differences in study design (longitudinal or cross-sectional), ethnicity, use of medications (iron, folic acid and multivitamins), susceptibility to medications etc.”

***Major comment 2.*** Not only did RLS improve for some of the subjects in this study, 100% of the subjects with RLS prior to pregnancy had resolution of RLS by the third trimester. This fact is still not adequately discussed by the authors, particularly as it varies so much from established literature.

**Response:**

We sincerely apologize for inadequate discussion regarding significant reduction in prevalence rates of idiopathic RLS from 2^nd^ to 3^rd^ trimester. We have added discussion regarding this point in the 3^rd^ paragraph of the Discussion (line 3-17, page 12) as follows.

“In the present analysis, prevalence of idiopathic RLS decreased from 3.3% in the 2^nd^ trimester to 0% in the 3^rd^ trimester. There has still be limited publications which reported trend in prevalence of RLS by type (idiopathic vs secondary) from 2^nd^ to 3^rd^ trimester. An observational study of 1584 pregnant women in China reported higher prevalence of idiopathic RLS in 3^rd^ trimester (5.2%) than in 2^nd^ trimester (4.3%) while this was a cross-sectional study and did not follow participants from 2^nd^ to 3^rd^ trimester. Another longitudinal study of 642 pregnant women in Italy reported comparable prevalence rates of idiopathic RLS between 2^nd^ and 3^rd^ trimesters but approximately 11% of participants with idiopathic RLS in the 2^nd^ trimester showed improvement in RLS symptoms in the 3^rd^ trimester. The reasons for various time courses in prevalence of idiopathic RLS in 2^nd^ and 3^rd^ trimesters are not clear but discrepancies might be attributable to differences in study design (longitudinal or cross-sectional), ethnicity, use of medications (iron, folic acid and multivitamins), susceptibility to medications etc.”

***Major comment 3.*** The statement re: the role of iron in RLS is simplistic and lacking a citation. The thought that use of iron supplements may account for some of the improvement in symptoms is reasonable, though that this may explain the large difference between the prevalence of this study and others is unconvincing as most countries have similar recommendations.

**Response:**

We apologize for lack of citation regarding lack of information regarding role of iron. We have now added reference 23,24 in the last sentence of the 2^nd^ paragraph of the Discussion (line 1, page 12).

We agree with you that iron supplements cannot explain large differences in prevalence of idiopathic RLS between our study and previous studies. At the moment, the reasons for various time courses in prevalence of idiopathic RLS in 2^nd^ and 3^rd^ trimesters are not clear but discrepancies might be attributable to differences in study design (longitudinal or cross-sectional), ethnicity, use of medications (iron, folic acid and multivitamins), susceptibility to medications etc. We have discussed this point in the 3^rd^ paragraph of the Discussion (line 3-17, page 12) as follows.

“In the present analysis, prevalence of idiopathic RLS decreased from 3.3% in the 2^nd^ trimester to 0% in the 3^rd^ trimester. There has still be limited publications which reported trend in prevalence of RLS by type (idiopathic vs secondary) from 2^nd^ to 3^rd^ trimester. An observational study of 1584 pregnant women in China reported higher prevalence of idiopathic RLS in 3^rd^ trimester (5.2%) than in 2^nd^ trimester (4.3%) while this was a cross-sectional study and did not follow participants from 2^nd^ to 3^rd^ trimester. Another longitudinal study of 642 pregnant women in Italy reported comparable prevalence rates of idiopathic RLS between 2^nd^ and 3^rd^ trimesters but approximately 11% of participants with idiopathic RLS in the 2^nd^ trimester showed improvement in RLS symptoms in the 3^rd^ trimester. The reasons for various time courses in prevalence of idiopathic RLS in 2^nd^ and 3^rd^ trimesters are not clear but discrepancies might be attributable to differences in study design (longitudinal or cross-sectional), ethnicity, use of medications (iron, folic acid and multivitamins), susceptibility to medications etc.”
